# Effect of α-Bisabolol and Its β-Cyclodextrin Complex as TetK and NorA Efflux Pump Inhibitors in *Staphylococcus aureus* Strains

**DOI:** 10.3390/antibiotics9010028

**Published:** 2020-01-14

**Authors:** Rafael Pereira da Cruz, Thiago Sampaio de Freitas, Maria do Socorro Costa, Antonia Thassya Lucas dos Santos, Fábia Ferreira Campina, Raimundo Luiz Silva Pereira, José Weverton Almeida Bezerra, Lucindo José Quintans-Júnior, Adriano Antunes De Souza Araújo, José Pinto De Siqueira Júnior, Marcello Iriti, Elena Maria Varoni, Irwin Rose Alencar De Menezes, Henrique Douglas Melo Coutinho, Maria Flaviana Bezerra Morais-Braga

**Affiliations:** 1 Department of Biological Sciences, Regional University of Cariri, Crato, Ceará 63105-000, Brazil; rafaelcruz284@gmail.com (R.P.d.C.); thiagocrato@hotmail.com (T.S.d.F.); corrinha_live@yahoo.com.br (M.d.S.C.); thassyalucas@hotmail.com (A.T.L.d.S.); fabiacampina@gmail.com (F.F.C.); raimundoluizbio@gmail.com (R.L.S.P.); weverton.almeida@urca.br (J.W.A.B.); flavianamoraisb@yahoo.com.br (M.F.B.M.-B.); 2 Department of Physiology, Federal University of Sergipe, São Cristóvão, Sergipe 49100-000, Brazil; lucindojr@gmail.com (L.J.Q.-J.); adriasa2001@yahoo.com.br (A.A.D.S.A.); 3 Department of Molecular Biology, Federal University of Paraíba, João Pessoa, Paraíba 58051-900, Brazil; jpsiq@uol.com.br; 4 Department of Agricultural and Environmental Sciences, Milan State University, 20133 Milan, Italy; marcello.iriti@unimi.it; 5 Department of Biomedical, Surgical and Dental Sciences, Milan State University, 20142 Milan, Italy; 6 Department of Biological Chemistry, Regional University of Cariri, Crato, Ceará 63105-000, Brazil; irwinalencar@yahoo.com.br (I.R.A.D.M.); hdmcoutinho@gmail.com (H.D.M.C.)

**Keywords:** resistance, antibiotics, sesquiterpene, bacteria

## Abstract

Efflux pumps are proteins present in the plasma membrane of bacteria, which transport antibiotics and other compounds into the extracellular medium, conferring resistance. The discovery of natural efflux pump inhibitors is a promising alternative. α-Bisabolol is a sesquiterpene isolated from several plants such as *Matricaria chamomilla* L. and has important properties such as antibacterial and anti-inflammatory activity. Currently, the formation of inclusion complexes with β-Cyclodextrin has been used for improving the physicochemical characteristics of the host molecule. This study evaluated the effect of α-Bisabolol, in isolation and in complexation with β-Cyclodextrin, as TetK and NorA efflux pump inhibitors in *Staphylococcus aureus* strains. The minimum inhibitory concentration (MIC) was determined. Subsequently, inhibitory activity over the pumps was observed by an MIC reduction for the antibiotics, by using subinhibitory concentrations (MIC/8) in combination with tetracycline and norfloxacin. The MIC of the compounds was ≥1024 μg/mL. α-Bisabolol potentiated the action of tetracycline and reduced the MIC of norfloxacin to a clinically relevant concentration. The complexed substance showed synergism however, the effect of the isolated α-Bisabolol was superior to the complex. These results indicate α-Bisabolol is a potential substance to be used as an efflux pump inhibitor.

## 1. Introduction

Bacterial resistance to chemotherapy represents a serious problem for human health. The indiscriminate use of antibiotics for the treatment of infections has enabled the emergence of mechanisms of resistance between various microbial strains, allowing the survival and spread of pathogenic bacteria. This problem has been aggravated, especially in hospital environments, and is directly related to high mortality numbers and medical expenses, reinforcing the inefficiency and limitations of the use of certain drugs [1,2].

The mechanisms enabling bacterial cells to resist the action of chemotherapeutic agents include: (a) alteration of the drug target sites, (b) enzymatic destruction of the antibiotic, (c) reduction in cell membrane permeability and (d) efflux pumps [3]. Efflux pumps have an important role in the emergence of multiple drug resistant (MDR) bacterial strains. *Pseudomonas aeruginosa*, *Escherichia coli*, and *Staphylococcus aureus* are bacteria of medical interest that use this survival strategy [4,5].

Efflux pumps are proteins integrated into the cell membrane and are responsible for pumping antibiotics and toxic substances into the extracellular medium [6]. Thus, efflux pumps reduce the concentration of these substances inside the cell, and consequently reduce the microorganism’s susceptibility to the drug’s effect [7]. Many Gram-positive and Gram-negative bacterial strains possess efflux pumps, and the genes encoding these proteins are located in chromosomes or plasmids [8,9].

Certain natural and synthetic substances have been previously described as efflux pump inhibitors (EPI’s), which are a therapeutic alternative against this resistance mechanism through their combination with antibiotics. Thus, a synergistic effect may reverse this resistance and restore drug efficacy against MDR bacteria. However, many of these characterized molecules present a high toxicity and low biological stability [10].

The search for new substances with pharmacological value from medicinal plant-derived secondary metabolites is a promising field [11,12]. Plants have a great chemical diversity with the potential of possessing antimicrobial agents, with their activity being previously proven and described as an important candidate for the development of new natural efflux pump inhibitors [13,14].

α-Bisabolol is a monocyclic sesquiterpene present in the essential oils of *Vanillosmopsis erythropappa* Sch. Bip. (Candeia), *Vanillosmopsis arborea* Barker (candeeiro) and *Matricaria chamomilla* L. Rausch (camomilla), belonging to the Asteraceae family, which may also be isolated from other aromatic plant varieties [15,16]. Following extraction, α-Bisabolol varies from colorless to light yellow, having a slightly viscous appearance and strong floral aroma. Because of this feature it is widely used for flavoring liquids and foods and is also present in preparations of makeup, eye creams, moisturizers, antiperspirants, cleansers, and sunscreens [17,18]. It also presents important biological and pharmacological properties, such as anti-inflammatory, antiparasitic, anti-tumor, anti-irritant, antiallergic, antifungal, and antibacterial activity [19,20,21,22].

One of the major limitations of using natural products is a reduced water solubility and bioavailability of the molecules of interest. An innovative strategy to overcome such challenges is their complexation with cyclodextrins; in other words, the formation of inclusion complexes to increase the water solubility of the plant compound [23]. This molecular property has enabled the use of cyclodextrins in different applications in science and technology, mainly in the pharmaceutical industry, by virtue of obtaining new drugs with different physical and chemical properties with the same active compounds [24].

β-CD is the most commonly used natural cyclodextrin in drug inclusion complex formation [25]. It has great complexation versatility with different hydrophobic drugs, the cavity size is ideal for drugs between 200 and 800 g/moL, it is easy to obtain at low financial cost, and its efficacy is approved for use [26].

In view of the above, the present study aims to evaluate the effect of the α-Bisabolol and β-Cyclodextrin natural compounds, both in isolation and through inclusion complexes, as inhibitors against *Staphylococcus aureus* strains carrying genes encoding an active efflux mechanism.

## 2. Results and Discussion

### 2.1. Antibacterial Activity

According to Table 1, α-Bisabolol and β-Cyclodextrin, both isolated and complexed, did not present direct antibacterial activity over *S. aureus* strains carrying efflux pumps. They had an MIC ≥ 1024 μg/mL, this concentration being considered clinically irrelevant [27]. Only the positive control (chlorpromazine) inhibited both strains at the 128 μg/mL concentration.

In the study by Oliveira et al. [28] the antimicrobial potential of the isolated α-Bisabolol and its complex with β-Cyclodextrin was evaluated against the *S. aureus* 10 multi-resistant strain. Both compounds did not present an efficacy (MIC ≥ 1024 μg/mL), corroborating with the present results showing these natural substances do not have a direct action against *S. aureus* strains expressing some types of resistance. However, this is the first study evaluating α-Bisabolol and its complex against efflux pump carrying strains.

In studies done by Andrade et al. and Oliveira et al. [28,29] similar results were found for the pure β-CD against *S. aureus, P. aeruginosa*, and *E. coli* bacteria, reinforcing the absence of an effect for this oligosaccharide against these strains.

### 2.2. Inhibitory Effect over Pumps by MIC Reduction

**Figure 1:**
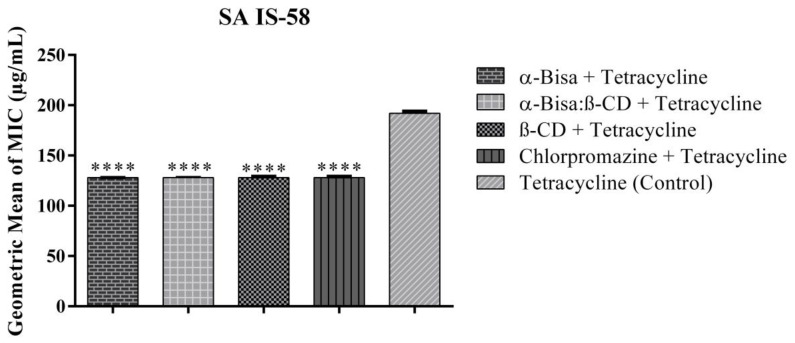
Effect of the compounds associated with tetracycline against *Staphylococcus aureus* IS-58. SA: *Staphylococcus aureus*, α-Bisa: Isolated α-Bisabolol, α-Bisa: β-CD: α-Bisabolol and β-Cyclodextrin inclusion complex, β-CD: β-Cyclodextrin. **** statistically significant value with *p* < 0.0001.

Although α-Bisabolol had no direct antimicrobial effect when tested at subinhibitory concentrations (MIC/8) when in combination with tetracycline and norfloxacin antibiotics, this sesquiterpene demonstrated a synergistic effect, that is, it potentiated the action of the drugs against the strains. When combined with tetracycline, all tested compounds presented synergism reducing the effective tetracycline concentration from 192 μg/mL to 128 μg/mL against the SA IS-58 TetK pump expressing strain (Figure 1).

Brehm–Stecher and Johnson [30] noted in their study that some sesquiterpenes such as nerolidol, farnesol, bisabolol, and apritone increased the susceptibility of *S. aureus* to several antibiotics, including tetracycline. The study was done with an experimental assay using the disc-diffusion method, where this effect may be due to a cell membrane disruptive action by the compounds which allowed an accumulation of drugs in the cytoplasm. This activity is most commonly evidenced in Gram-positive bacteria, perhaps because they have fewer membranes than Gram-negative bacteria. The increased permeability of sesquiterpenes may be due to its structural similarity to the lipid membrane, thus enhancing the spectrum of action of antibiotics [30].

**Figure 2:**
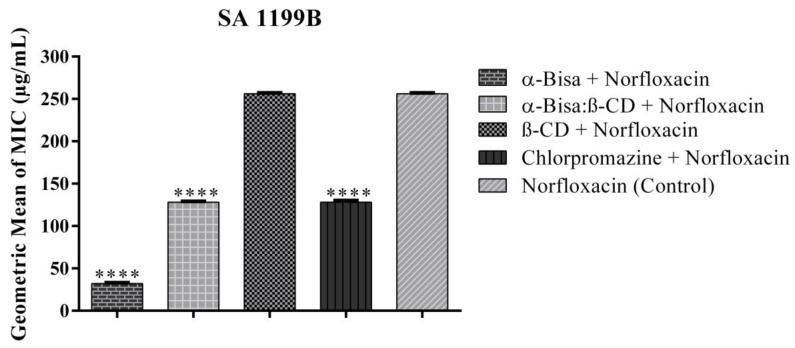
Effect of the compounds associated with norfloxacin against *Staphylococcus aureus* 1199B. SA: *Staphylococcus aureus*, α-Bisa: isolated α-Bisabolol, α-Bisa: β-CD: α-Bisabolol and β-Cyclodextrin inclusion complex, β-CD: β-Cyclodextrin. **** statistically significant value with *p* < 0.0001.

For the SA 1199 B strain, α-Bisabolol in combination with norfloxacin reduced the antibiotic’s MIC from 256 μg/mL to 32 μg/mL, showing significant synergistic activity (Figure 2). α-Bisabolol presented a better modulatory effect than chlorpromazine—an EPI from the phenothiazines class that has been widely used to sensitize bacteria resistant to various antibiotics and to promote the accumulation of ethidium bromide [31,32]—thus demonstrating potential as a possible NorA pump inhibitor that expels hydrophilic fluroquinolones. The mechanisms of inhibition in MDR bacteria are not yet fully understood. However, it is assumed the inhibitor binds to the pump and blocks its function in a competitive or noncompetitive manner to the substrate or that it causes disruption in the energy source required for extrusion [33].

A synergistic interaction between α-Bisabolol and norfloxacin was also reported for the SA 10 strain in the study by Oliveira et al. [28]. Other terpenes and essential oils are described as potentiators in *S. aureus* efflux pump carrying strains. The essential oil from *Chenopodium ambrosioides* L. leaves, a mixture rich in terpenes, was tested against SA IS-58 strains. When associated with tetracholine it interacted synergistically, thus being attributable to its phytochemical constituents [34]. Some sesquiterpene derivatives isolated from the *Ferula ferulioides* K. plant presented a potentiating effect for norfloxacin against the SA-1199B NorA pump expressing strain [35].

Due to the lipophilic character of terpenes, a greater ease of proton and ion penetration exists in the cell membrane, which may cause modifications in the function and structure of proteins, including efflux proteins from many infectious microorganisms [36,37]. Although the α-Bisa: β-CD inclusion complex reduced the MIC of Norfloxacin, the effect of the isolated α-Bisabolol has been shown to be greater than that of the complex and is capable of inhibiting the NorA pump at a much lower concentration. This is because the inclusion complex can modify the physicochemical characteristics of the host substance and promote structural reorganization of the molecule, interfering in the interaction of the compound and consequently its bioactivity [38]. Conversely, the complex at the 1:1 molar ratio may not have been enough to improve the physical characteristics of the molecule. Waleczek et al. [39] analyzed the stability of the α-Bisabolol/β-CD complex and found that α-Bisabolol was not completely included in β-CD, suggesting that an increase in β-CD quantity (2:1) may improve complexation.

## 3. Materials and Methods

### 3.1. Bioactive Compounds and Complexation

The α-Bisabolol and β-Cyclodextrin compounds were obtained from Sigma-Aldrich^®^, San Luis, MO, USA. The formation of the α-Bisabolol/β-CD inclusion complex was performed and donated by the Pharmacy Laboratory of the Federal University of Sergipe (UFS). First the substances were mixed and 2 mL of distilled water was added. The substances were homogenized with a mortar and pistil. Afterwards complexation occurred at a 1:1 molar ratio (222.36 g·moL^−1^: 1135 g·moL^−1^) in 20 mL of water under constant stirring over a 36 h period. The samples were desiccated and removed by manual grinding. The results from the complex characterization are described in the study by Oliveira et al. [28]. For the microbiological assays, 10 mg of each compound were weighed and diluted in 1 mL dimethyl sulfoxide (DMSO), then a second dilution in sterile distilled water was performed until the initial concentration of 1024 μg/mL was reached.

### 3.2. Culture Media

The following culture media were used for microbiological tests: Heart Infusion Agar (HIA, laboratories Difco Ltda., Detroit, MI, USA) prepared according to the manufacturer and Brain Heart Infusion (BHI Acumedia Manufacturers, Inc.) prepared to a concentration of 10%.

### 3.3. Bacterial Strains

The *S. aureus* bacterial strains used were: IS-58, endowed with plasmid pT181 carrying the TetK efflux pump protein that extrudes tetracycline; and the 1199B strain that presents resistance to norfloxacin by NorA pump expression. The strains were donated to the Laboratory of Microbiology and Molecular Biology (LMBM) by Prof. S. Gibbons (University of London), where they were cultured and kept in stock at 4 °C in solid Heart Infusion Agar (HIA, Difco) medium. The IS-58 plasmid carrier strain was seeded in culture medium with the antibiotic tetracycline at a sub-inhibitory concentration in order to guarantee gene expression and to avoid losing this characteristic.

### 3.4. Drugs, Pump Inhibitors, and Reagents

The antibiotics tetracycline and norfloxacin, as well as the efflux pump inhibitor chlorpromazine, were diluted in distilled sterile water to a concentration of 1024 μg/mL. The resazurin sodium colorimetric bacterial growth indicator (through oxide reduction) was used for the test readings.

### 3.5. Determination of the Minimum Inhibitory Concentration (MIC)

The minimum inhibitory concentration test was performed by the broth microdilution method [40]. The stock strains were seeded in HIA solid medium and kept in an incubator at 37 °C for a period of 24 h. Thereafter, with the aid of a nickel-chromium loop, colonies of replicate cells were collected and inoculated into test tubes containing 3 mL of sterile saline solution (0.9%). The inocula were compared to the Mcfarland scale 10^5^ CFU. After standardization, Eppendorfs^®^ containing 1350 μL of the BHI liquid culture medium and 150 μL of the bacterial inoculum were prepared. Subsequently, microdilution plates were filled with 100 μL of the total content solution from the Eppendorfs^®^. Microdilution was then performed, where 100 μL of each compound was added into the first well and serial dilutions (1:1) were carried out up to the penultimate cavity, with the last one being used as the growth control. The concentration of the compounds ranged from 512 to 0.5 μg/mL. At the end of the experiment the plates were incubated in a bacteriological oven at 37 °C for 24 h. 20 μL of sodium resazurin was added to the wells for reading and after one hour any color changes in the wells were noted, where a change from blue to pink indicated colony growth. The MIC was defined as the lowest concentration where bacterial growth was not observed according to the CLSI (Clinical and Laboratory Standards Institute) [41].

### 3.6. Evaluation of Efflux Pump Inhibition by a Modulating Effect

MIC reduction is a method used to identify possible efflux pump inhibitors [42]. To check the modulatory effect of the antibiotic plus natural compound combination on efflux pump inhibition, the method performed by Coutinho et al. [43] was used, where the compounds were evaluated at sub-inhibitory concentrations (MIC/8). For this purpose, Eppendorfs^®^ were filled with 150 μL of the bacterial inoculum, with all compounds at sub-inhibitory concentrations (MIC/8), and were supplemented with BHI liquid culture medium until it reached 1.5 mL in volume. A modulation control was also prepared with the same inoculum quantity and 1350 μL of BHI. Next, the plates were filled and micro diluted with 100 μL of the antibiotics up to the penultimate cavity (1:1). The plates were incubated and readings with resazurin were taken. Antibiotic MIC reduction when in modulation with chlorpromazine is an indication of the presence and inhibition of efflux pumps.

### 3.7. Statistical Analysis

The tests were performed in triplicate and the results expressed as mean ± standard deviation, using GraphPad Prism software version 6.0. The results were subjected to a one-way (ANOVA) followed by Bonferroni’s post hoc test, where the results were considered significant when *p* < 0.05.

## 4. Conclusions

The compounds tested did not present a direct antibacterial activity against the efflux pump carrying strains, presenting MIC values at clinically irrelevant concentrations. However, the isolated α-Bisabolol potentiated the action of the antibiotics against the tested strains, with the α-Bisabolol/β-CD inclusion complex also presenting synergism in a more moderate manner. This provided evidence for the complex changes important properties and the mode of action of the compound. The α-Bisabolol mechanisms of interaction with the efflux pumps are not yet sufficiently known. Further studies are needed to prove the potential of the compound as a possible inhibitor and to unmask if the observed effect is due to membrane rupture or substrate ligation and blockage of the H+ energy source. Investigating the formulation of these substances is thus necessary for future applications in the improvement of antibiotics in the fight against bacterial resistance. In any case, the use of mutant strains of SA 1199B with no efflux pump expression will certainly corroborate these preliminary findings, as well as RT-PCR analysis to investigate the gene expression levels of NorA and TetK genes after treatment.

## Figures and Tables

**Table 1:** Determination of the minimum inhibitory concentration (μg/mL) of α-Bisabolol, α-Bisabolol/β-CD, β-Cyclodextrin, and Chlorpromazine.

Strain	SA IS-58	SA 1199B
α-Bisabolol	≥1024	≥1024
α-Bisabolol/β-CD	≥1024	≥1024
β-Cyclodextrin	≥1024	≥1024
Chlorpromazine	128	128

**Legend:** SA: *Staphylococcus aureus*, α-Bisabolol/β-CD: α-Bisabolol and β-Cyclodextrin inclusion complex.
